# Which Performance Tests Best Define the Special Judo Fitness Test Classification in Elite Judo Athletes?

**DOI:** 10.3390/jfmk7040101

**Published:** 2022-11-14

**Authors:** Bayram Ceylan, Jožef Šimenko, Şükrü Serdar Balcı

**Affiliations:** 1 Department of Coaching Education, Faculty of Sport Sciences, Kastamonu University, Kastamonu 37150, Türkiye; 2 School of Life and Medical Sciences, University of Hertfordshire, Hatfield AL10 9EU, UK; 3 Department of Coaching Education, Faculty of Sport Sciences, Selcuk University, Konya 42130, Türkiye

**Keywords:** performance evaluation, SJFT index, combat sports, field testing

## Abstract

The normative values of the Special Judo Fitness Test (SJFT) are used to evaluate judo athletes, and the question arises of which performance tests from crucial motor abilities best define the SJFT classification in elite judo athletes. This study aimed to investigate the relationship between elite judo athletes’ physical performance and the evaluation using SJFT index norms. Nineteen judo athletes (11 females) (22.8 ± 2.5 years old) from the senior judo national team voluntarily participated in this study. Body composition, reaction time, balance, flexibility, agility, hand grip strength, 20 m sprint, vertical jump, SJFT, and Wingate tests were performed by athletes on four separate days at one-day intervals. Athletes were classified as regular and above (≥regular) or poor and below (≤poor) according to their SJFT index scores. Simple logistic regression analysis was used to evaluate this classification’s consistency with performance test results. Odds ratios (OR) and 95% confidence intervals related to each possible factor and Wald test statistics were calculated. The SJFT index classification was associated with Wingate peak and mean power, vertical jump, and sprint performance results (*p* < 0.05), whereas it was not associated with body fat percentage, agility, reaction time, hand grip strength, flexibility, and balance performances (*p* > 0.05). SJFT index classificatory norms are mainly related to athletes’ anaerobic power. Higher anaerobic power increases athletes’ possibility of being classified as ≥regular.

## 1. Introduction 

The complexity of judo, with numerous throws, ground techniques, technical–tactical skills, and considerable coordinative demands [1], affects the coaches’ training design so their athletes obtain the best results in competitions [2]. To monitor the judokas’ physical readiness and training progress, coaches and researchers use one of the most widely used field tests in judo, the Special Judo Fitness Test (SJFT) [3,4,5]. The SJFT formula is based on a total of throws (TT) made in three sets (1–15 s, 2–30 s, 3–30 s) separated by a 10 s break and heart rate (HR) collected immediately after the test and after 1 min with the inversely related index as the primary outcome [6]. 

The SJFT was shown to be a reliable test [7] that can be used for training monitoring in junior and senior athletes [8,9] and can differentiate between regional, national [10], and high-level judokas [11,12,13]. SJFT test has also been used to detect movement asymmetries [14,15], and its index was also highly correlated to the number of attacks during a judo match [16] and with laboratory performance tests like the Wingate test and graded exercise treadmill tests [17,18,19]. 

The actual applicable use of the SJFT index for the coaches was done in 2009 with the senior SJFT male classificatory tables [20] and later in 2014 for females with critical division into senior and junior female judokas [21]. In addition, male and female cadet and junior classificatory tables were added to the literature in 2018 [22], and in 2019, male senior and junior classificatory tables were updated with an extensive meta-analysis [23]. The SJFT presents a simple and widely available method and is one of the most frequently used tests in judo. The negative aspects that coaches and researchers often present about the SJFT involve running and the usage of only one technique (ippon seoi nage) from 68 Nage-waza techniques [24]. The use of Ippon seoi nage could be defended as it is one of the most frequently and widely used throws in judo fights [25]. Additionally, it has been reported that it is uncomfortable for uke (judokas being thrown), which needs to be from the same weight category as the tori (judoka performing the test), and it does not differentiate between weight categories [24]. 

The factors influencing success in judo, according to Sertić & Lindi [26], are coordination (15%), strength (22%), speed (12%), mobility (8%), balance (8%), personality traits (10%), mental abilities (10%), and cardiorespiratory abilities (15%). Other authors have also connected the ability to differentiate movements, speed, accuracy, the precision of movements, sports level, and complex reaction time to influence success in judo [27]. Additionally, factors like relative strength, topological factor, flexibility, absolute dynamic strength, and the ability to perform complex motor tasks of a speed-explosive type were identified to influence performance in judo [28]. There is a lack of studies that would comprehensively test multiple performance variables to the SJFT index and its classificatory tables. The question arises of which performance tests from crucial motor abilities best define the Special Judo Fitness Test classification in elite judo athletes. Therefore, the main aim of our study was to find out which performance tests best define the Special Judo Fitness Test classification in elite judo athletes. 

## 2. Materials and Methods

### 2.1. Study Design

The measurements were carried out at the end of the preparation camp prior to the competitive period of the athletes. Following a 14-day training camp, athletes were given a day for recovery, and measurements were implemented after athletes were informed about the test procedures and measurements in detail. The measurements were performed on four separate visits with a one-day interval in between. During the first visit, body composition, reaction time, agility, handgrip strength, 20 m sprint, and vertical jump were measured. On the second visit, balance and flexibility tests were carried out. A special judo fitness test (SJFT) was implemented on the third visit. The fourth and final visit included the performance of the 30 s Wingate test. All the measurements were performed between 09:00–11:00 in the morning. Also, there was a rest interval of at least 10 min between consecutive tests on the same day.

Women [21] and men [23] were classified as ≥regular and ≤poor according to the SJFT index calculated with total throw numbers and heart rate during SJFT. The compatibility between this classification and athletes’ physical performances was examined. Z-scores of similar tests and total scores related to those variables were calculated [29]. The formula given below (*a*) was used to calculate z-score. The formula was revised if the performance decreased when the test score increased (*b*).

$$\left(a\right)\;Z-score=\frac{X_{1}\;\bar{X}}{SD}\;\;\left(b\right)\;Z-score=\frac{\bar{X}-X_{1}}{SD}$$

### 2.2. Participants

Nineteen elite senior judo athletes (8 male; 11 female) (age = 22.8 ± 2.5) from the Turkish National Team participating in international competitions for Olympic qualification voluntarily participated in this study. The study’s ethical approval was obtained from the Kastamonu University Ethical Committee (Number: 2020-KAEK-143-74). The study was implemented according to the latest version of the Declaration of Helsinki. Because these elite-level athletes participate in such measurements throughout the year, they were familiar with the procedures, but they were informed about the study and all measurements in detail, and a signed informed consent form was obtained. The measurements were carried out in the judo hall and laboratory. 

### 2.3. Body Composition

A calibrated scale (Seca 876, Seca Ltd., Birmingham, UK) was used to measure the athletes’ body mass with a 0.1 kg precision, and a portable stadiometer (Seca, Hamburg, Germany) was used to measure their height with a 1 mm accuracy. The body mass index was calculated using the formula BMI = body mass (kg)/height (m)^2^. To calculate body fat percentage (BFP), a skinfold caliper (Holtain, UK) with 10 g/sq mm pressure at each angle was used. Four sites—the biceps, triceps, subscapular, and suprailiac—were used to measure skinfold. Durnin and Womersley’s equation [30] was used to compute body density, while Siri’s equation was used to calculate body fat percentage. A qualified and experienced person carried out the measurements. 

### 2.4. Reaction Time Test

The Optojump-Next Microgate tool (Optojump, Bolzano, Italy) and program were used to test reaction time (Microgate, Italy). The athletes, standing in a semi-squat position (approximately 120–140°) with their hands on the waist and feet at shoulder width, were asked to jump as soon as possible with the visual stimulus coming from the computer. In the reaction test, the interval between the arrival of a visual stimulus on the computer screen and a jump was measured and recorded as athletes’ performance. With at least 15 s of rest between each repetition, this procedure was repeated two times, and the best result was taken for further analysis [31]. 

### 2.5. Balance Test

A balance-measuring device (TechnoBody PK 252 platform, Dalmine, Italy) with a 20-Hz sampling rate and sensitivity of 0.1° was used to measure the static and dynamic balance performances of the athletes. Both tests were implemented in a bipedal posture with eyes open on a stationary platform. Participants stood shoulder-width apart on the balancing platform, equidistant from the origin point as measured by the x- and y-axis lines. To calculate static balance scores, participants were required to hold their stance for 30 s while watching their status information on a screen. The participants stared at a fixed point in front of them, and after they were balanced, the test was started manually and ended automatically by the computer. Summing the units of the medial-lateral and backward-forward deviations from the center point provided the static balance test score. The pressure level was adjusted to a difficulty level of five to test dynamic balance (out of 50). The participants were asked to control their posture to move the cursor along the middle circle accurately 5 times as quickly as possible in one minute. The average track error data were computed as a percentage of the departure from the center point and utilized to generate the dynamic balance score. In both tests, greater values indicated poor balance, and lower ones suggested excellent balance [32,33]. Before each test, the descriptive data of the subjects was input, and the equipment was calibrated. The dynamic and static balancing tests were each repeated twice, with the better test result being used in the analysis.

### 2.6. Flexibility Test

The sit-and-reach test was conducted using a specifically designed box. A centimeter scale was placed on the top of the box’s surface. The athlete was instructed to sit with straight legs and reach as far forward as they could while sliding their hands over the surface with their palms down, placing one hand on top of another. They held that position for roughly 6 s. The athlete’s final position was measured in centimeters. Athletes had three attempts with 30-s rest, and the highest value was recorded for further analysis. 

An active knee extension test was used to measure hamstring muscle length. Before taking measurements, a physiotherapist indicated the center of the knee joint axis above the right leg’s lateral joint line. Two lines were drawn from this location, one connecting the axis point to the center of the femur’s greater trochanter and the other connecting the axis point to the apex of the lateral malleolus. After testing, the lines were erased with alcohol and repainted at the start of each testing session. The participant lay in a supine position on a bench and flexed their right knee and hip to 90 degrees. They were advised not to allow the femur to migrate away from the hand at any stage throughout the test and to monitor its location with their right hand. The participant was told to stretch their right leg as far as they could while keeping their foot relaxed and to hold the posture for 5 s. Each participant did a single repeat of the activity to become acquainted with it. A second repeat was conducted, and the angle of knee extension was measured using a standard Perspex goniometer (Physiomed, Manchester, UK) at the end of the 5 s. The goniometer’s center was placed over the axis point previously marked on the lateral joint line, and the goniometer arms were placed along the femur and fibula lines. To ensure that each individual had the same length of static stretch, the goniometer measurement was obtained within 2 s of reaching the end range of knee extension [34]. As for shoulder internal-external rotation, a 12-inch, 360° goniometer with two movable overlapping arms marked in 11 increments was utilized. Standard measurement positioning was used [35] during the whole process. Shoulder external rotation was obtained by passively putting the athlete’s arm in 90 degrees abduction with the elbow flexed 90 degrees and requesting the athlete to rotate their arm back as far as possible such that their palm faced the ceiling. The stationary arm was placed perpendicular to the floor, and the moving arm was aligned with the shaft of the ulna and the styloid process. Shoulder internal rotation was obtained by passively putting the athlete’s arm in 90 degrees abduction with the elbow bent 90 degrees and requesting the athlete to rotate their arm forward as far as possible such that their palm faced the floor. The total flexibility score was obtained by calculating the z-score of each variable.

### 2.7. Agility Test (t-Test)

The test comprised of four spots within a 10 m long and 10 m wide area. The participant sprinted or moved as swiftly as possible to the center cone on the “go” order, then sidestepped to the right 5 m to the right cone, to the left 10 m to the far left cone, and finally to the center cone by sidestepping back to the right. The athlete then ran back as fast as they could to cross the finish line. The test duration was monitored with a photocell (Newtest Powertimer 300-series, Newtest Oy, Tyrnävä, Finland) and recorded in seconds [36]. 

### 2.8. Handgrip Strength 

A handgrip dynamometer was used to assess handgrip strength in a standing posture three times on each side alternately, with 1 min intervals between efforts (Takei Scientific Instruments CO., Tokyo, Japan). Participants randomly chose the starting hand. Athletes were asked to create the greatest force in 3–5 s while fully extended elbows and self-selected wrist and leg postures were taken. For the analysis, the highest value for each side was utilized [37]. 

### 2.9. 20 Meter Sprint Test

The wireless photocell (Newtest Powertimer 300-series, Newtest Oy, Tyrnävä, Finland) was used for the measurement. The athlete tried to run as fast as possible and reach the finish line with the “go” starting command by the researcher. The time was recorded in seconds. Athletes performed two trials with at least 3 min rest in between, and the best performance was recorded for further analysis.

### 2.10. Special Judo Fitness Test

Following a 5 min standardized warm-up comprising running, judo falling techniques (ukemi), and repeating throwing techniques without falling, three athletes of comparable body weight and height conducted the SJFT according to the following protocol: two judokas (uke) were placed 6 m apart to be thrown, while the test executor (tori) was placed, in the middle, 3 m apart from the judokas. The procedure consisted of three sessions of 15 s (A), 30 s (B), and 30 s (C), with 10-s intervals between them. During each session, the executor threw the opponents as many times as possible, utilizing the ippon seoi nage technique. The total number of throws completed for each of the three sessions (A + B + C) was used to measure performance. The heart rate (HR) was monitored immediately following the test and again 1 min later (SEEGO RealTrack, Spain) to determine the index using the equation:

$$\mathrm{Index}\;(\mathrm{bpm}.{\mathrm{throws}}^{-1})=\frac{\mathrm{final}\;\mathrm{HR}\;(\mathrm{bpm})+\mathrm{HR}\;\mathrm{at}\;1\;\min\;\mathrm{after}\;\mathrm{the}\;\mathrm{test}\;\left(\mathrm{bpm}\right)}{\mathrm{Number}\;\mathrm{of}\;\mathrm{throws}}$$

### 2.11. Wingate Anaerobic Test

The test was implemented on a cycle ergometer (Monark Ergomedic 894 E, Monark, Sweden). As the athletes had performed this test many times previously, a familiarization session was not provided. Each athlete’s seat height was modified. Participants began with a 5 min warm-up at an unloaded resistance. The test resistance was estimated using 75 g per kilogram for each athlete’s body mass. Athletes were instructed to pedal as fast as possible for 30 s, and athletes remained seated throughout the test. Also, athletes were provided with verbal encouragement during the test. Peak power (PP) and mean power (MP) were recorded [38,39]. 

### 2.12. Statistical Analysis

The mean, standard deviation, and 95% confidence intervals (CI) of the data were presented. Normality was checked with the Shapiro-Wilk test as well as descriptive methods using skewness and kurtosis coefficients [40]. The linear correlation between SJFT index scores and physical performance tests was investigated using Pearson Correlation analysis. There is 1 variable per 10 events criterion for binary logistic regression analysis [41]. However, as the study group was composed of Olympic judo team athletes, the number of participants was limited. Therefore, the analysis evaluated each performance test as a separate model. A simple logistic regression was implemented to confirm the effect of physical performance tests on the athletes’ SJFT index classification (i.e., ≥regular or ≤poor). Odds ratios (OR) and 95% confidence intervals related to each possibility factor were calculated using Wald test statistics. Brier score was used to evaluate the predictive accuracy of binary prediction models [42]. The Brier score range for overall accuracy is 0 and 1, with the best possible Brier score being zero. The effect size for correlation analysis was presented as small (0.10), medium (0.30), and large (0.50) according to the correlation coefficient (r) [43]. Statistical analysis was carried out with JASP 0.16.3 software. Significance was set as *p* < 0.05.

## 3. Results

Physical characteristics and performance test results of the elite judo athletes are presented in Table 1.

The simple logistic regression results regarding the effect of elite athletes’ performances on SJFT index classification (i.e., ≥regular or ≤poor) can be seen in Table 2. Although there was a significant correlation between body fat percentage and SJFT index score (r = 0.81; *p* < 0.001, ES = Large), it was not related to SJFT index classification (Cox and Snell R2 = 0.62; β = −0.98; SE(β) = 0.61; Wald χ2(1) = 2.62; *p* = 0.11). There was a significant correlation between the 20 m sprint test results and the SJFT index (r = 0.74; *p* < 0.001, ES = Large). Moreover, sprint time is related to SJFT index classification (Cox and Snell R2 = 0.48; β = −10.43; SE(β) = 5.14; Wald χ2(1) = 4.11; *p* = 0.04). While the athletes’ agility performance duration decreased, their SJFT performance increased (r = 0.77; *p* < 0.001, ES = Large). Nevertheless, athletes’ agility performance was not related to SJFT classification (Cox and Snell R2 = 0.21; β = −1.36; SE(β) = 0.77; Wald χ2(1) = 3.14; *p* = 0.08). There was no correlation between handgrip strength and SJFT index scores (r = −0.18; *p* = 0.47, ES = Small), and in line with this finding, it was not related to SJFT classification (Cox and Snell R2 = 0.20; β = 1.37; SE(β) = 0.08; Wald χ2(1) = 3.13; *p* = 0.08). There was a negative correlation between vertical jump and SJFT index scores (r = −0.74; *p* < 0.001, ES = Large). A one-unit increase in vertical jump increased the possibility of athletes’ being classified higher than regular 1.6 times (Cox and Snell R2= 0.56; β =0.44; SE(β) = 0.20; Wald χ2(1) = 4.77; *p* = 0.03). While the correlation was not significant between the SJFT index and peak power (r = −0.53; *p* = 0.08, ES = Large), there was a significant correlation between the SJFT index and mean power (r = −0.72; *p* < 0.001, ES = Large). A one-unit increase in peak power increased the possibility of athletes’ success during SJFT almost 4 times (OR = 4.37) (Cox and Snell R2 = 0.42; β = 1.48; SE(β) = 0.71; Wald χ2(1) = 4.29; *p* = 0.04), while mean power increased almost 9 times (OR = 8.57) (Cox and Snell R2 = 0.40; β = 2.15; SE(β) = 1.06; Wald χ2(1) = 4.12; *p* = 0.04). There was no significant correlation between the fatigue index and the SJFT index (r = −0.36; *p* = 0.14, ES = Medium). Fatigue index was not related to SJFT classification (Cox and Snell R2 = 0.09; β = 0.07; SE(β) = 0.06; Wald χ2(1) = 1.33; *p* = 0.25). There was no significant correlation between reaction time (r = 0.43; *p* = 0.07, ES = Medium), total flexibility (r = 0.09; *p* = 0.71, ES = Small), and total balance scores (r = −0.33; *p* = 0.16, ES = Medium) and SJFT index. Moreover, reaction time (Cox and Snell R2 = 0.11; β = −10.52; SE(β) = 7.58; Wald χ2(1) = 1.93; *p* = 0.17), total flexibility score (Cox and Snell R2 < 0.01; β = 0.01; SE(β) = 0.03; Wald χ2(1) = 0.06; *p* = 0.80) and total balance score (Cox and Snell R2 = 0.03; β = −0.02; SE(β) = 0.03; Wald χ2(1) = 0.49; *p* = 0.48) were not related to SJFT classification. 

## 4. Discussion

This is the first study investigating the relationship between physical fitness tests and the classification tables specifically developed for SJFT. A relationship has been known between the SJFT index and general performance test results [6,19,44,45]. Based on this information, it is foreseen that the important performance elements for judo are highly associated with SJFT classificatory table classifications. Although this hypothesis was partially approved for peak and mean power obtained via the Wingate test, vertical jump, and 20 m sprint test, no relationship was presented related to body fat percentage, agility, reaction time, hand grip strength, flexibility, and balance performances. The main finding of the current study was that athletes with higher anaerobic power had more possibility to be classified as ≥regular according to the SJFT classification table. Even though there was a large effect size relationship between SJFT index and body fat percentage and agility performance, these variables were not related to the classification (i.e., poor-regular) in the classificatory table.

SJFT, which can be applied easily in a judo hall, is a practical test developed to assess judo athletes’ physical fitness [4]. Although it has been frequently used in studies related to judo athletes’ performance, the first classificatory table was developed from the data of 141 male judo athletes aged between 16–34 with five scales (20% for each classificatory category) [20]. However, Sterkowicz-Przybycień and Fukuda [21] developed norm values with five scales (Excellent = highest 5%, Good = next highest 15%, Regular = middle 60%, Poor = next lowest 15%, and Very poor= lowest 5%) via meta-analysis using 96 female athletes ‘data. Therefore, a direct comparison of the SJFT index between genders is not recommended as it does not follow the same methodology. However, later Sterkowicz-Przybycień et al. [23] developed a revised classificatory table for male athletes using 515 adult judo athletes’ data via the same methodological approach with meta-analysis. Although the latest two studies considered age and sex factors while creating norm values, the differences that may stem from weight categories were neglected. Previous studies have already highlighted this problem and suggested that body mass should be considered in the SJFT interpretation because heavier athletes achieve worse results than lighter athletes, but they can have similar competitive success in their respective weight divisions [44]. Moreover, there is no information related to the homogeneity of the athletes’ weight classes in the studies implemented to develop SJFT norm tables. This increases the questions on the reliability of the SJFT index tables. 

In the current study, 47.4% of the athletes were classified as ≤poor, while 52.6% were classified as ≥regular. Of the athletes who participated in the current study, 26% were heavyweight, 10.5% were middleweight, 5.3% were half-middleweight, 26.3% were lightweight, 15.8% were half-lightweight, and 15.8% were extra-lightweight. It is known that judo athletes’ physical structures and motor performances [46] and technical and tactical elements [47] are different. Although the classifications mentioned above were developed by taking age and sex into account, the most important limitation was not neglecting the weight category factor. In the current study, correlations with high effect sizes were determined between SJFT index scores and athletes’ alactic anaerobic powers, i.e., peak power, 20 m sprint, and vertical jump. Moreover, athletes with high anaerobic power are more likely to be classified as ≥regular, according to the SJFT classificatory table. The dominant energy system used during SJFT is the alactic anaerobic system, and it was reported that this test could assess athletes’ alactic anaerobic power performance [17]. It can be said that athletes’ performance test results related to the dominant energy system during SJFT are consistent with the classificatory table. Although Paulo Lopes-Silva et al. [48] indicated that physical fitness parameters obtained from upper and lower body aerobic and anaerobic tests had a small ability to predict SJFT variables, the results of the current study showed that SJFT classificatory table is associated with anaerobic power variables.

Although significant positive correlations with large effect sizes were determined between SJFT index score, body fat percentage, and agility test duration, they had no relationship with the classification in the SJFT classification table. Reaction time, flexibility, and balance performances were not associated with both the SJFT index score or the classification in the table. Previous research reported significant correlations between the SJFT index and body composition, agility, strength, reaction time, flexibility, fatigue index, and balance performances [45,49]. The inconsistent results from different studies may have stemmed from numerous reasons, such as the number of participants [22,50], nonhomogeneous levels of the athletes [20], and methodological differences while applying the tests. According to the classificatory table, a relationship between performance tests and SJFT index scores cannot guarantee athletes’ classification. In this context, it can be said that SJFT norms are only associated with anaerobic power. 

Although SJFT is known to successfully discriminate athletes from different levels [51,52], it was not associated with the technical-tactical performance of the athletes during official competitions [53,54]. Related to its ability to discriminate athletes from different competitive categories (i.e., elite, and non-elite), SJFT was expected to reflect other physical performance test results as well as anaerobic performance. However, the findings of this study could not approve this hypothesis. This may have stemmed from the fact that many complex motoric and psychological abilities affect judo performance. Also, there have been numerous changes in judo competition rules and thus match duration and content since the development of SJFT [55,56,57,58,59]. In line with these changes, judo athletes’ physiological characteristics have also changed [60]. Moreover, although SJFT has been used for almost 30 years, it has not changed; a classificatory table without considering weight categories is a vital deficiency [8]. The special techniques athletes use, 6 m sprinting distance component and 15-30-30 s work periods, could also be revised in the future. 

The most important limitation of the current study was the sample size. The small sample size may have increased Type I errors. However, the participants were composed of Olympic team judo athletes from the senior age category. Therefore, a larger sample size with an equal number of male and female athletes with separate evaluations of sexes could strengthen the suggestions in the future. 

## 5. Conclusions

The current study showed that the SJFT index classificatory norms are associated with anaerobic power, and higher anaerobic power increased the possibility of athletes’ classification as ≥regular. Nevertheless, there was no relationship between the classification in the SJFT classificatory table and athletes’ body composition, agility, strength, balance, and flexibility performances. These results showed that the SJFT classificatory table makes a classification related to athletes’ anaerobic power. The fact that sex and age categories were considered, and weight categories were neglected in the classification tables may have affected the results. Therefore, the norm values should be revised in the future according to weight categories, and their relationship with athletes’ physical performance should be re-evaluated.

## Figures and Tables

**Table 1:** Judo athletes’ physical characteristics and exercise tests performance.

	Mean ± SD	95% CI		
		Lower	-	Upper
Body height (cm)	167.4 ± 9.4	162.9	-	171.9
Body weight (kg)	79.1 ± 25.4	66.8	-	91.3
Body mass index (kg/m^2^)	27.8 ± 6.9	24.4	-	31.1
Body fat percent (%)	18.8 ± 7.6	15.1	-	22.4
SJFT total throw number	25.9 ± 3.2	24.4	-	27.4
SJFT index (point)	13.8 ± 1.8	12.9	-	14.7
Dominant hand grip strength (kg)	38.2 ± 8.2	34.3	-	42.2
20 m sprint (s)	3.3 ± 0.3	3.2	-	3.4
Agility (s)	9.8 ± 0.9	9.4	-	10.3
Vertical jump height (cm)	34.6 ± 8.3	30.6	-	38.6
Wingate peak power (W/kg)	10.8 ± 1.9	9.9	-	11.7
Wingate mean power (W/kg)	7.4 ± 1.3	6.7	-	8.0
Fatigue index (%)	55.3 ± 10.2	50.3	-	60.4
Reaction time for legs (s)	0.5 ± 0.1	0.5	-	0.6
Sit and reach (cm)	33.9 ± 10.9	28.7	-	39.2
Active knee extension (degrees)	8.3 ± 5.4	5.7	-	10.9
Shoulder internal rotation (degrees)	45 ± 8.5	40.9	-	49.1
Shoulder external rotation (degrees)	98.4 ± 6.6	95.2	-	101.6
Static balance anterior-posterior	−1.1 ± 1.9	−2.0	-	−0.2
Static balance medial-lateral	0.1 ± 1.1	−0.5	-	0.6
Dynamic balance (%)	20.7 ± 15.6	13.2	-	28.2

SD = standard deviation; CI = confidence interval; ATE = average track errors.

**Table 2:** Results from simple logistic regression model for the SJFT index classificatory table (≤Poor/≥Regular), showing Odds Ratios (OR) and their 95% confidence intervals (Cl), regression coefficients (β), and their standard errors (SE).

		≤Poor/≥Regular			
SJFT Index	*β*	SE *β*	Wald	*p*	OR (95%—CI)
Body fat percent (%)	−0.98	0.61	2.62	0.11	0.37 (0.11—1.23)
*(Brier score = 0.07, accuracy = 89.5%, sensitivity = 90.0%), specificity = 89.0%)*					
20 m sprint (s)	−10.43	5.14	4.11	0.04	0.00 (0.00—0.71)
*(Brier score = 0.13, accuracy = 84.0%, sensitivity = 90.0%), specificity = 78.0%)*					
Agility (s)	−1.36	0.77	3.14	0.08	0.26 (0.06—1.16)
*(Brier score = 0.19, accuracy = 73.7%, sensitivity = 80.0%), specificity = 66.7%)*					
Hand grip strength (kg)	1.37	0.08	3.13	0.08	1.15 (0.99—1.33)
*(Brier score = 0.20, accuracy = 68.4%, sensitivity = 80.0%), specificity = 55.6%)*					
Vertical jump (cm)	0.44	0.20	4.77	0.03	1.56 (1.05—2.32)
*(Brier score = 0.09, accuracy = 89.5%, sensitivity = 90.0%), specificity = 88.9%)*					
Wingate PP (watt/kg)	1.48	0.71	4.29	0.04	4.37 (1.08—17.67)
*(Brier score = 0.14, accuracy = 78.9%, sensitivity = 80.0%), specificity = 77.8%)*					
Wingate MP (watt/kg)	2.15	1.06	4.12	0.04	8.57 (1.07—68.47)
*(Brier score = 0.15, accuracy = 73.7%, sensitivity = 80.0%), specificity = 66.7%)*					
Fatigue index (%)	0.07	0.06	1.33	0.25	1.07 (0.95—1.20)
*(Brier score = 0.23, accuracy = 55.6%, sensitivity = 57.1%), specificity = 25.7%)*					
Reaction time (s) with both legs	−10.52	7.58	1.93	0.17	0.00 (0.00—76.23)
*(Brier score = 0.22, accuracy = 63.2%, sensitivity = 60.0%), specificity = 66.7%)*					
Total flexibility score	0.01	0.03	0.06	0.80	1.01 (0.95—1.06)
*(Brier score = 0.25, accuracy = 47.4%, sensitivity = 80.0%), specificity = 11.1%)*					
Total balance score	−0.02	0.03	0.49	0.48	0.98 (0.93—1.03)
*(Brier score = 0.24, accuracy = 63.2%, sensitivity = 70.0%), specificity = 55.6%)*					

*Hosmer and Lemeshow test, p > 0.05 for all the models. CM jump = Counter moment jump. Wingate PP = Wingate test peak power. Wingate MP = Wingate test mean power.*

## Data Availability

The data supporting this study’s findings are available from the authors upon reasonable request by a qualified researcher.
